# Supplementary material for: Wide-energy programmable microwave plasma-ionization for high-coverage mass spectrometry analysis
Source: Nat Commun. 2024 Jul 18;15:6075. doi: 10.1038/s41467-024-50322-z (PMC11258349; doi:10.1038/s41467-024-50322-z)
Supplement: Supplementary file 1 — Supplementary Information [file 41467_2024_50322_MOESM1_ESM.pdf]

Supplementary Information

Wide-energy programmable microwave plasma ionization for high-coverage mass spectrometry analysis

Fengjian Chu[1, 2], Gaosheng Zhao[3], Wei Wei[2], Nazifi Sani Shuaibu[1], Hongru Feng[2]*, Yuanjiang Pan[2]*, Xiaozhi Wang[1]*

[1]College of Information Science and Electronic Engineering, Zhejiang University, Hangzhou 310027, China;
[2]Department of Chemistry, Zhejiang University, Hangzhou 310027, China;
[3]School of Environmental and Chemical Engineering, Shanghai University, Shanghai 200444, China;

*Correspondence to: fenghongru@zju.edu.cn, panyuanjiang@zju.edu.cn, xw224@zju.edu.cn.

Table of Contents

1. Wide-energy programmable microwave plasma ionization mass spectrometry system: instrumentation and performance 3

2. Molecular structure and elemental analysis capabilities........ 5

3. Various application scenarios 17

4. Comparison and safety issues 22

5. Experimental section 24

Suppl. Table 5. Mass error of the products 26

Supplementary References 28

Supplementary Note 1

Wide-energy programmable microwave plasma ionization mass spectrometry system: instrumentation and performance

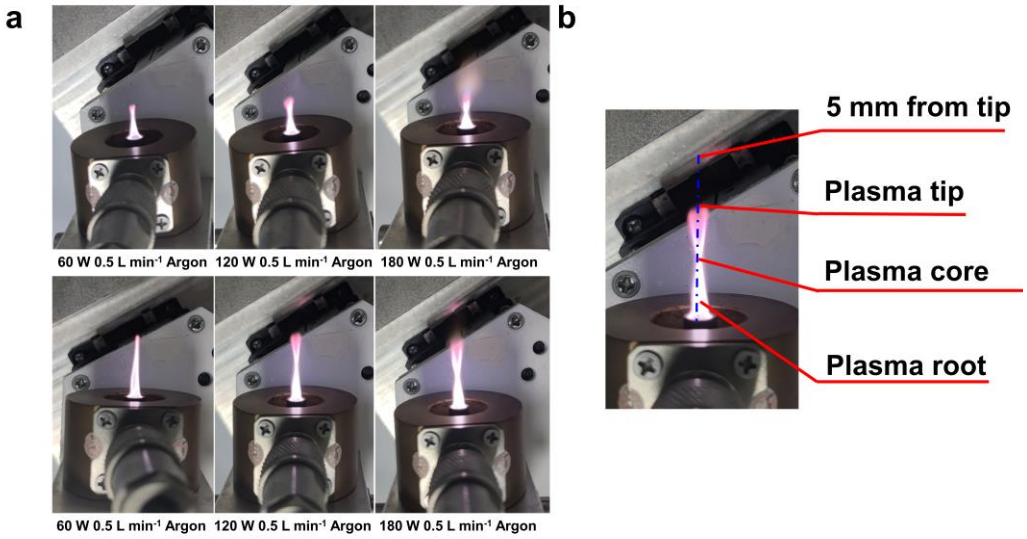

Supplementary Fig. 1. Plasma morphology diagram. a Plasma length and shape are affected by different microwave power and carrier gas flow. **b** Plasma thermometry sites, including the first 5 mm of the tip, the plasma tip, the plasma core and the plasma root.

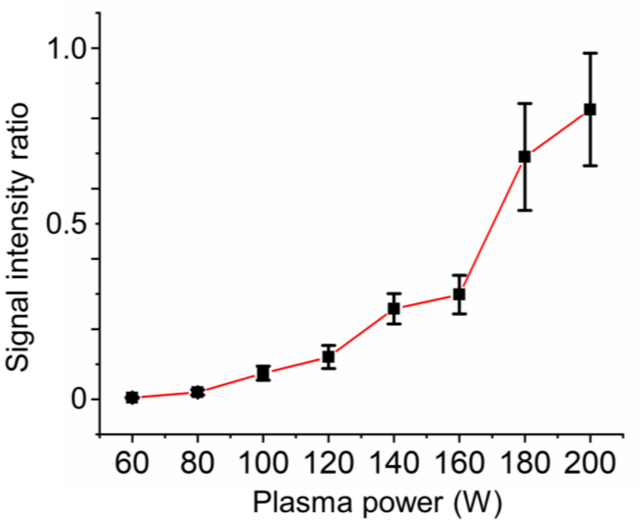

Supplementary Fig. 2. The ratio of methyl salicylate radical cation ion's intensity to protonated ion's intensity increases with increasing microwave plasma power, with a gentle change up to 160 W. Each value represents the mean ± s.d. ($N = 5$).

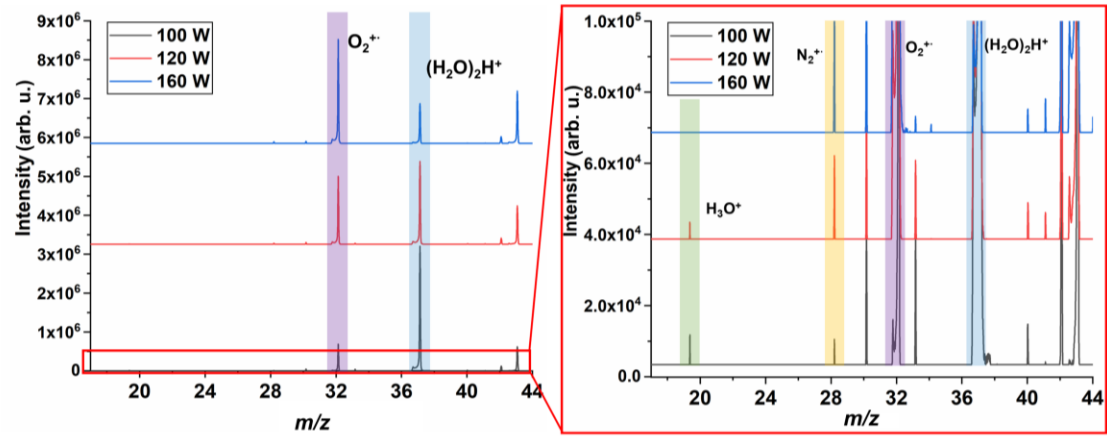

Supplementary Fig. 3. The populations of reagent ions shift in relative abundance with shifting power. Hydronium ions ($(H_2O)_nH^+$, green & blue), which cause proton transfer, decrease significantly with increasing power; while reagent ion species that can bring molecular ions, such as oxygen radicals (purple) nitrogen radical ions (yellow), increase in abundance.

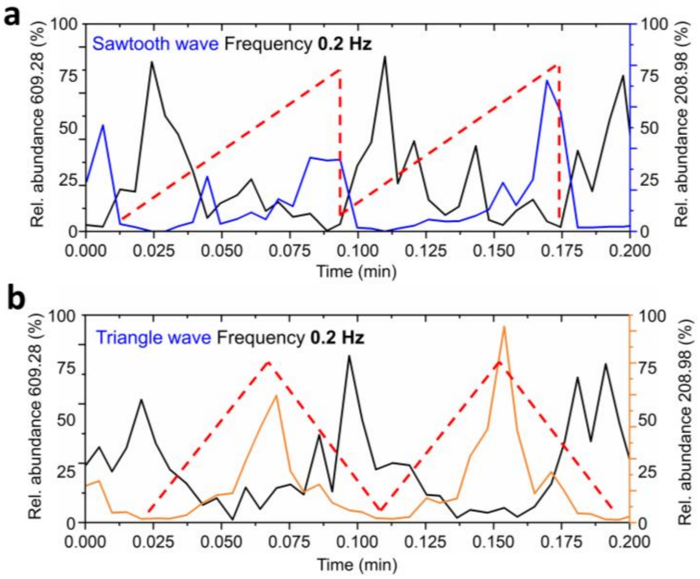

Supplementary Fig. 4. EIC plots of reserpine and divalent lead when ionized with different scanning waveforms (100-200 W) a EIC plots for both ions when sawtooth wave at 0.2 Hz is applied, the blue line represents the EIC plot of divalent lead ions within 0.2 min, and the black is the EIC plot of reserpine. **b** EIC plots when triangle wave at 0.2 Hz is applied, the orange line represents the EIC plot of divalent lead ions within 0.2 min, and the black is the EIC plot of reserpine.

Supplementary Note 2

Molecular structure and elemental analysis capabilities

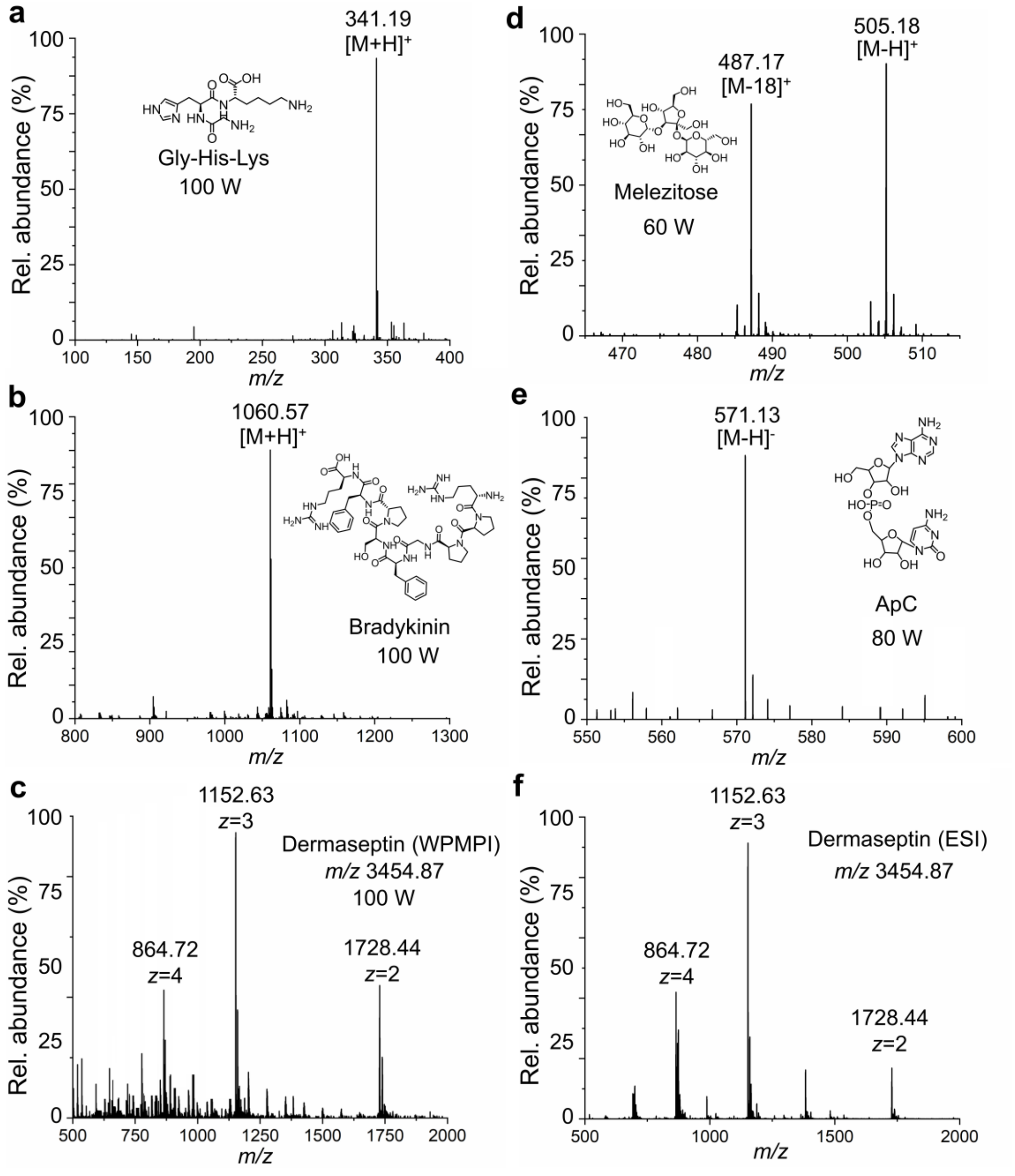

Supplementary Fig. 5. Mass spectra of 5 representative analytes in WPMPI-MS a Protonated ion mass spectra of Gly-His-Lys in the low-energy region of WPMPI-MS. **b** Protonated ion mass spectra of bradykinin in the low-energy region of WPMPI-MS. **c** Protonated ion mass spectra of dermaseptin in the low-energy region of WPMPI-MS, multiple charge state signals appear. **d** Protonated ion mass spectra of melezitose in the low-energy region of WPMPI-MS. **e** Deprotonated ion mass spectra of ApC in the low-energy region of WPMPI-MS. **f** ESI control mass spectra of dermaseptin.

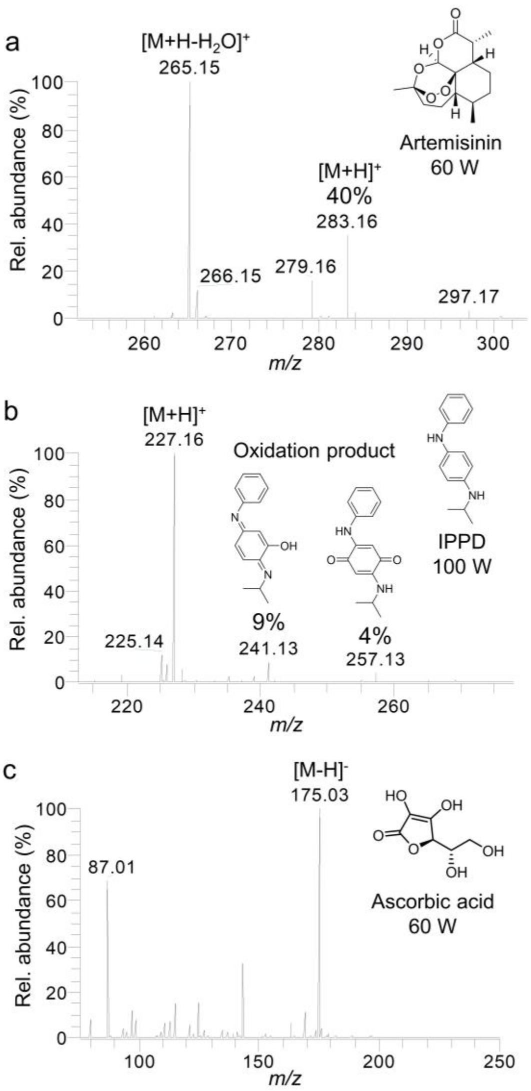

Supplementary Fig. 6. a WPMPI-MS with 60 W energy to analyze the artemisinin solution, abundant protonated molecular ions were observed (40%). **b** The easily oxidizable compound *N*-isopropyl-*N′*-phenyl-1,4-phenylenediamine (IPPD) ionized using WPMIP at 100 W. **c** The easily oxidizable compound ascorbic acid ionized using WPMIP at 60 W.

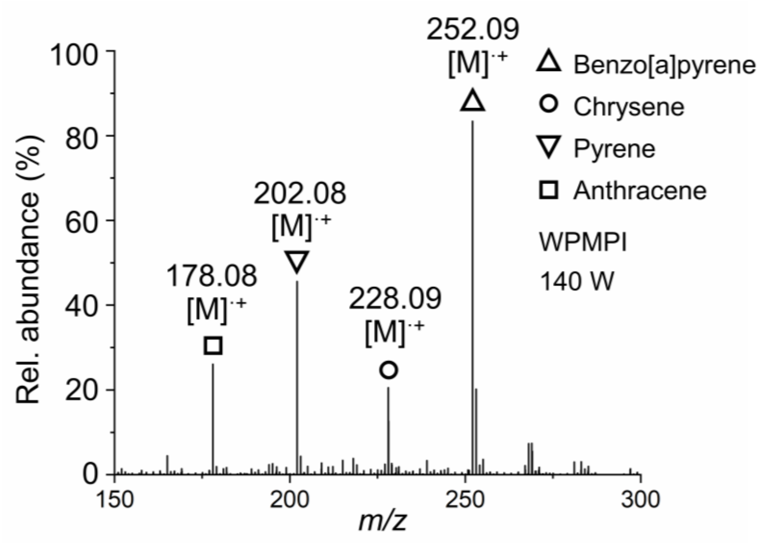

Supplementary Fig. 7. Four representative PAHs (anthracene, pyrene, chrysene, and benzo(a)pyrene) were analyzed by WPMPI-MS with the energy scanning interval in 140-180 W, the product ions are all radical molecular ions $[M]^{\cdot+}$.

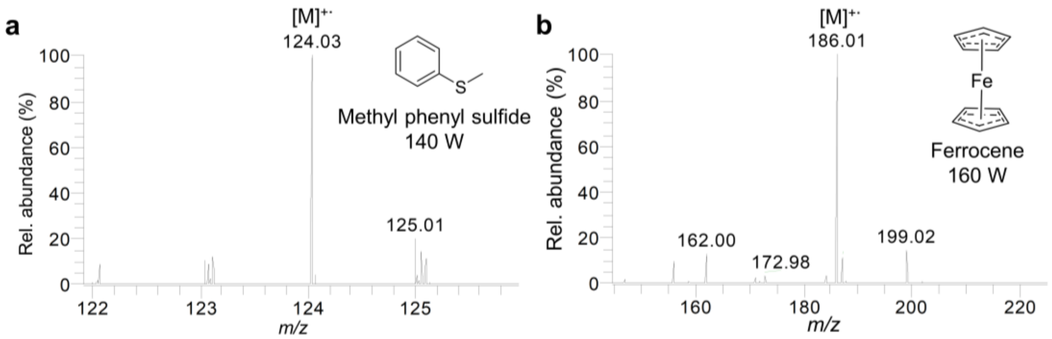

Supplementary Fig. 8. WPMPI mass spectra of low/non-polar analytes in methanol solution (10 mg L^{-1}): **a** methyl phenyl sulfide (*m*/*z* 124.03); **b** ferrocene (*m*/*z* 186.01).

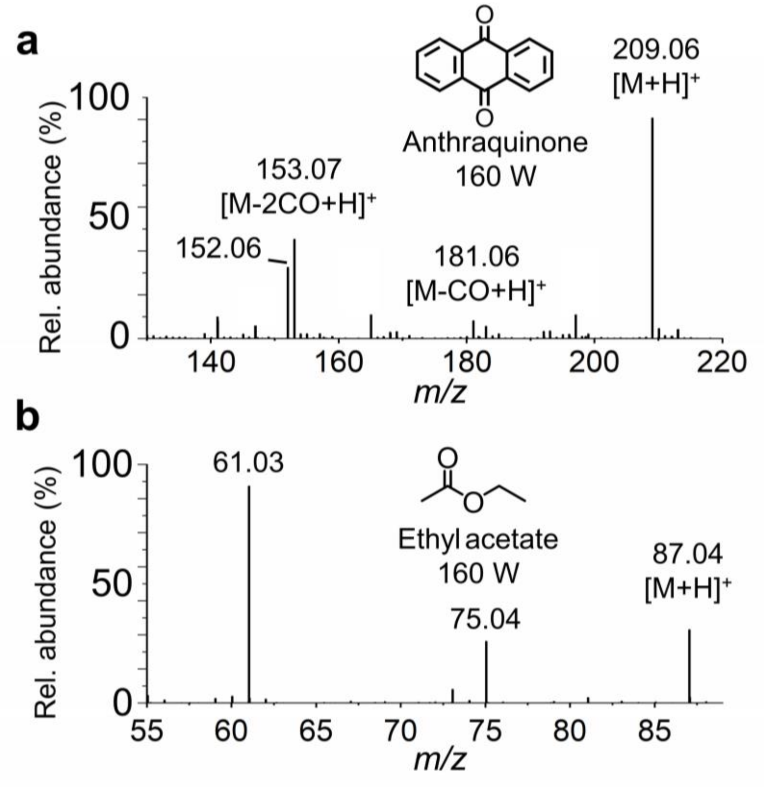

Supplementary Fig. 9. **a** Mass spectra of anthraquinone undergoing an elimination reaction in WPMPI-MS under atmospheric pressure, anthraquinone lost small molecules (one or two CO, *m*/*z* 181.06 and *m*/*z* 153.07). **b** Mass spectra of ethyl acetate undergoing an McLafferty rearrangement in WPMPI-MS under atmospheric pressure, the product ion *m*/*z* 61.03 was observed.

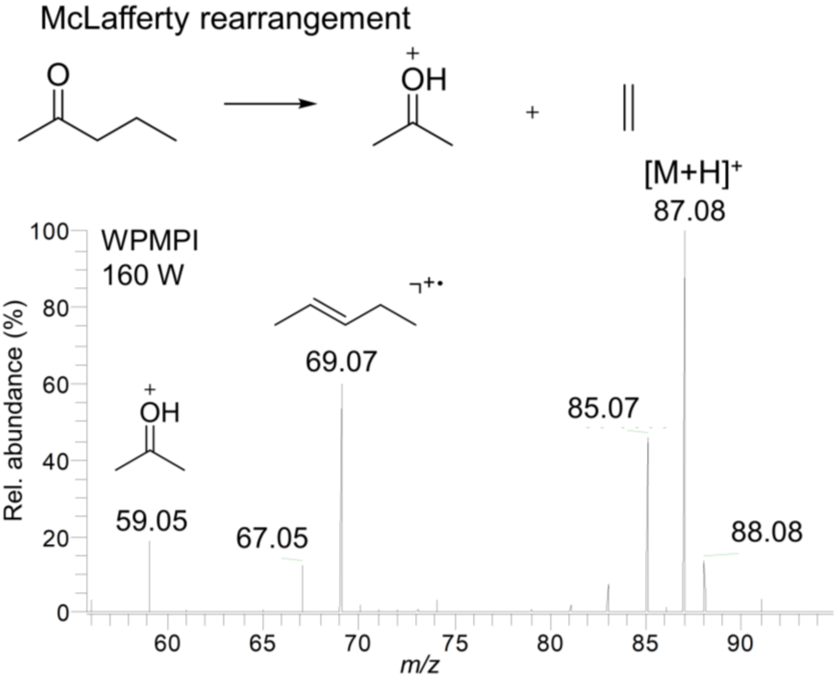

Supplementary Fig. 10. Mass spectra of 2-pentanone undergoing a McLafferty rearrangement reaction in WPMPI-MS under atmospheric pressure, with the rearrangement product ion at *m*/*z* 59.05.

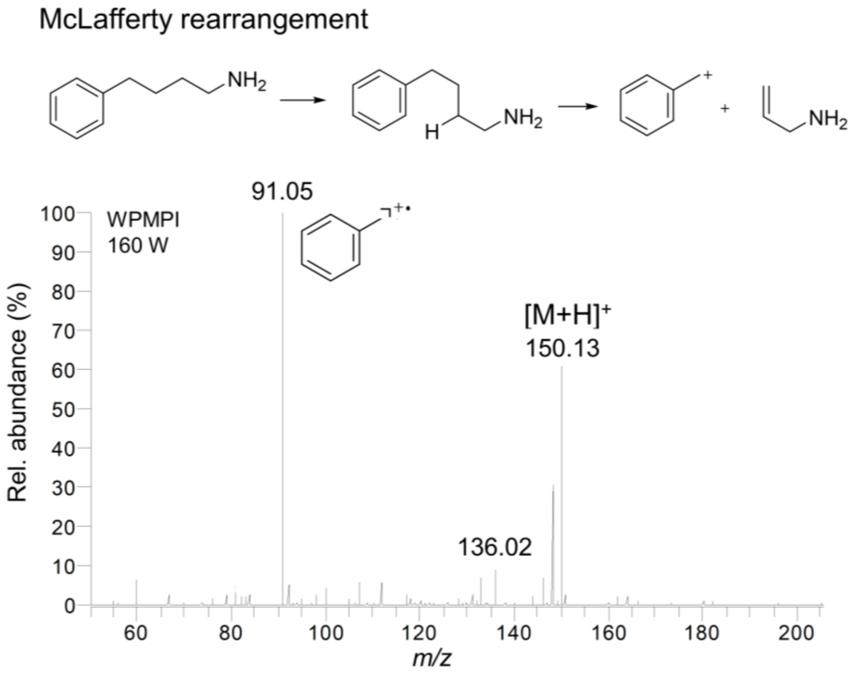

Supplementary Fig. 11. Mass spectra of 4-phenylbutylamine undergoing a McLafferty rearrangement reaction in WPMPI-MS under atmospheric pressure, with the rearrangement product ion at *m*/*z* 91.05.

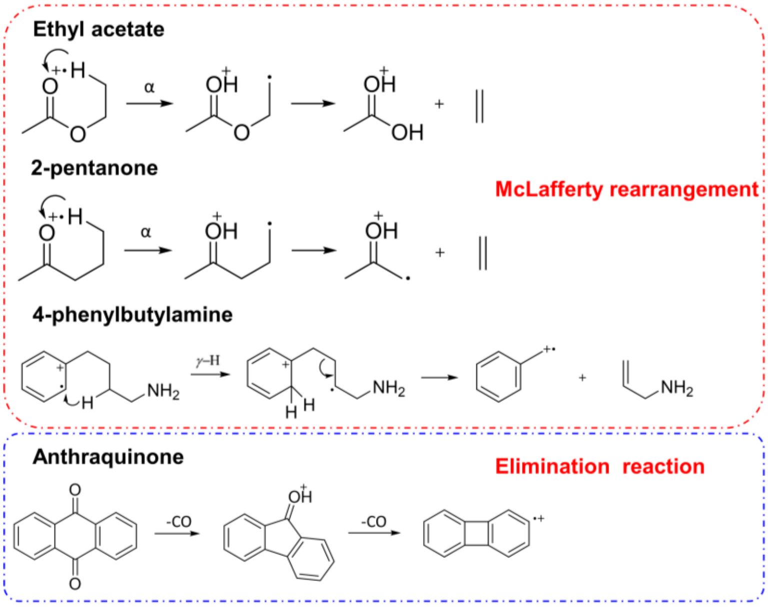

Supplementary Fig. 12. A mechanistic explanation of the EI-like rearrangement reactions occurring in WPMPI-MS under atmospheric pressure, including ethyl acetate, 2-pentanone, 4-phenylbutylamine that undergoes a Maillard rearrangement, to anthraquinone, which has an elimination reaction.

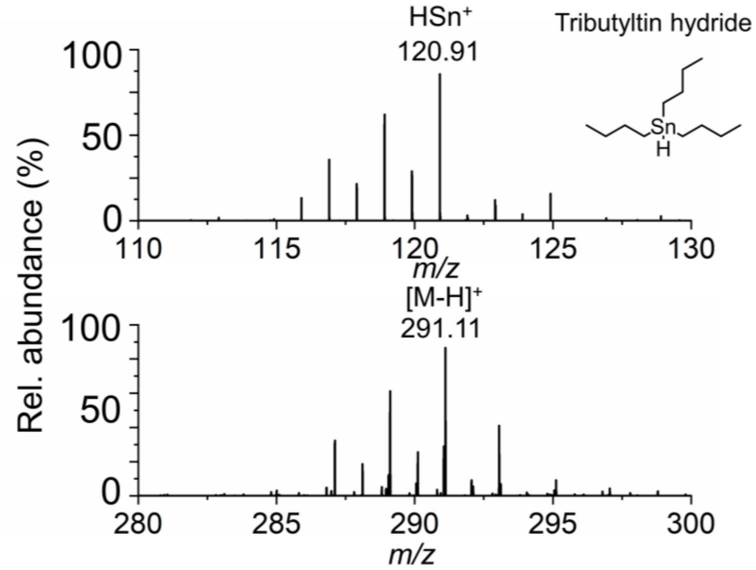

Supplementary Fig. 13. Isotope mass spectra of tributyltin hydride and resolved tin ions, where *m*/*z* 120.91 is the highest abundance isotope ions for tin monomers and *m*/*z* 291.11 is the tributyltin hydride deprotonation ions.

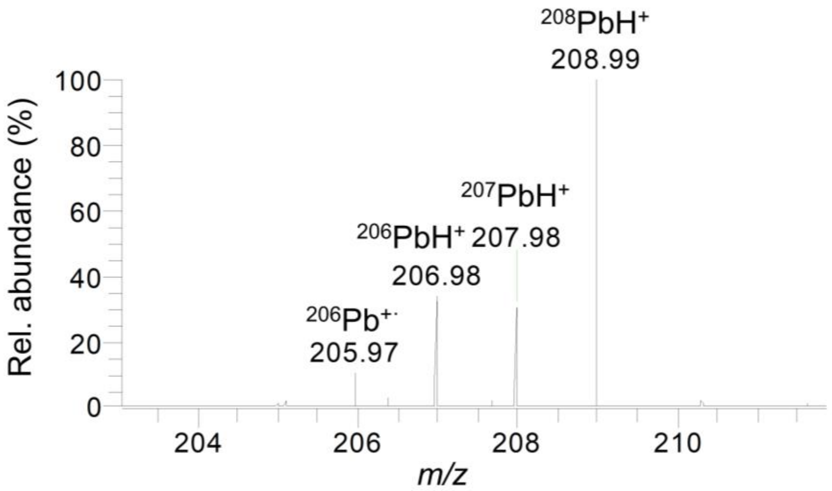

Supplementary Fig. 14. Isotope mass spectra of of divalent lead, where *m*/*z* 208.99 is the highest abundance isotope ions $^{208}PbH^{+}$, *m*/*z* 207.98 is $^{207}PbH^{+}$ ions, *m*/*z* 206.98 is $^{206}PbH^{+}$ ions.

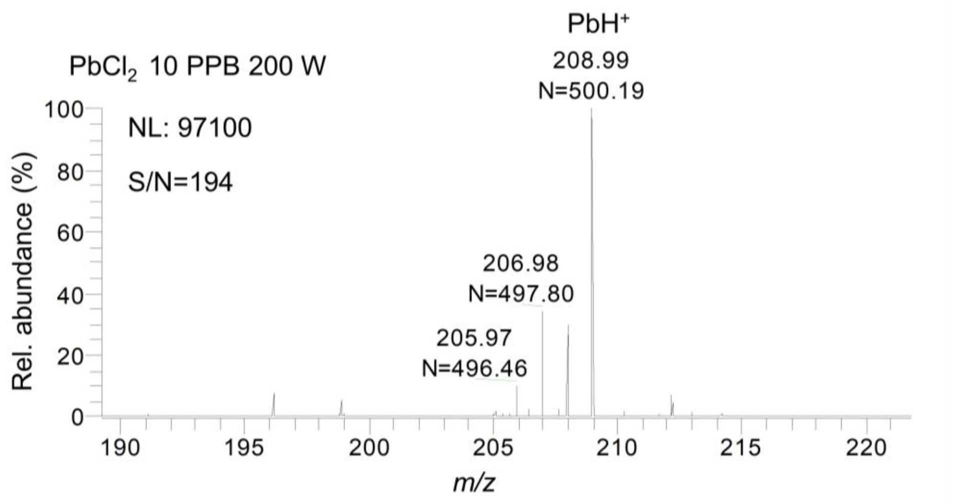

Supplementary Fig. 15. The signal-to-noise ratio of the WPMPI-MS was 194 (LOD ≈ sub ppt) when detecting lead chloride at a concentration of 10 ppb.

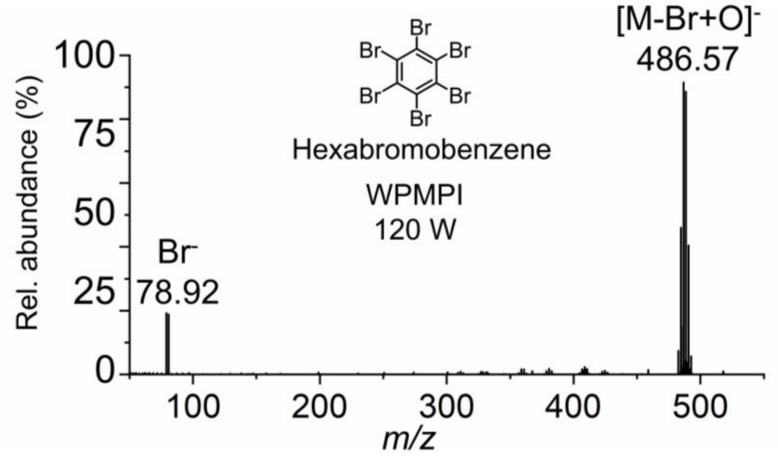

Supplementary Fig. 16. Mass spectra of hexabromobenzene in WPMPI-MS, except for the *m*/*z* 486.57 is the ionization product of hexabromobenzene, $[M\text{-}Br+O]^-$ (oxygen may come from atmospheric oxygen or nitro molecules), and in the same mass spectrogram, a clear isotopic signal of bromine monomers can be observed, *m/z* 78.92 (from dissociated hexabromobenzene).

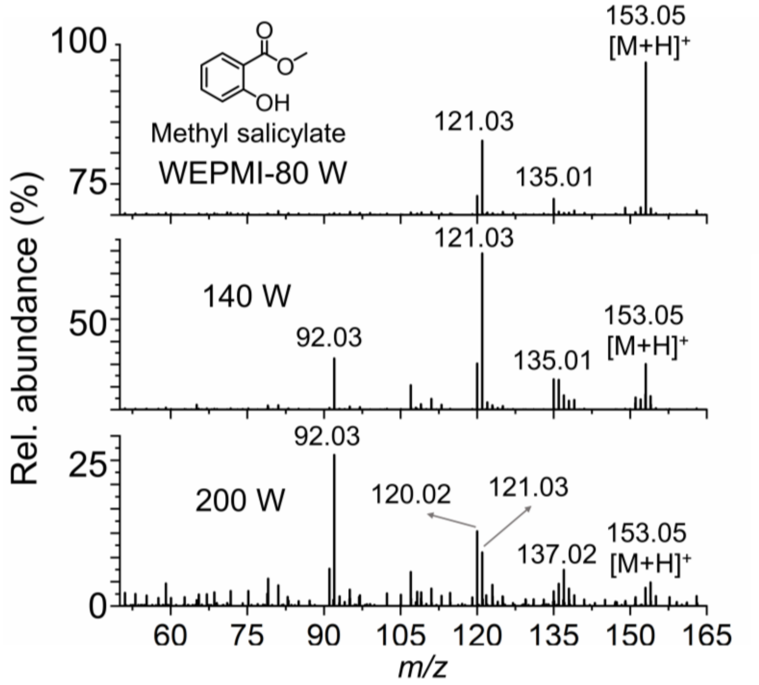

Supplementary Fig. 17. The fragment ions of methyl salicylate changed in species and abundance with increasing scanning power from 80 to 200 W. Among the fragment ions, *m*/*z* 121.03 and *m*/*z* 153.05 are the even-electron ion, *m*/*z* 92.03, *m*/*z* 120.03, and *m*/*z* 152.05 are odd-electron ions.

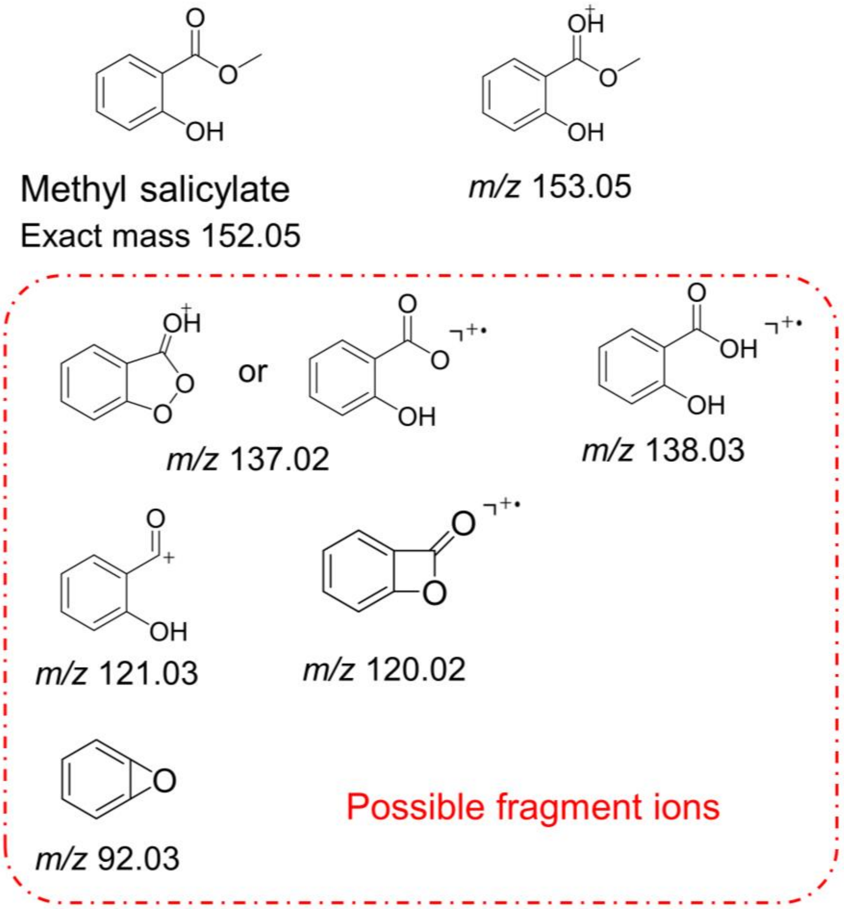

Supplementary Fig. 18. Structural inference of fragment ions produced by methyl salicylate in the presence of WPMPI-MS with different energy powers, which is consistent with the fragmentation pattern of arc plasma-based dissociation (APD)[1].

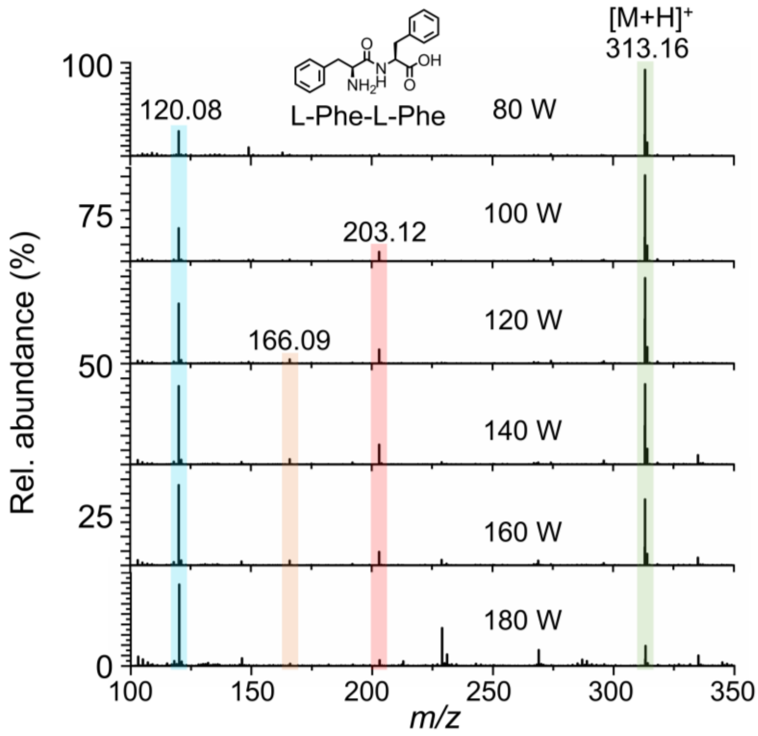

Supplementary Fig. 19. Dissociation spectrogram of phe-phe at different WPMPI power steps. Among the fragment ions, *m*/*z* 313.16 is the protonated ion of the dipeptide (green), and the percentage of fragment *m*/*z* 120.08 (blue) rises with increasing microwave power, while the abundance of two fragment ions, *m*/*z* 166.09 (orange) and *m*/*z* 203.12 (red), first increases and then decreases.

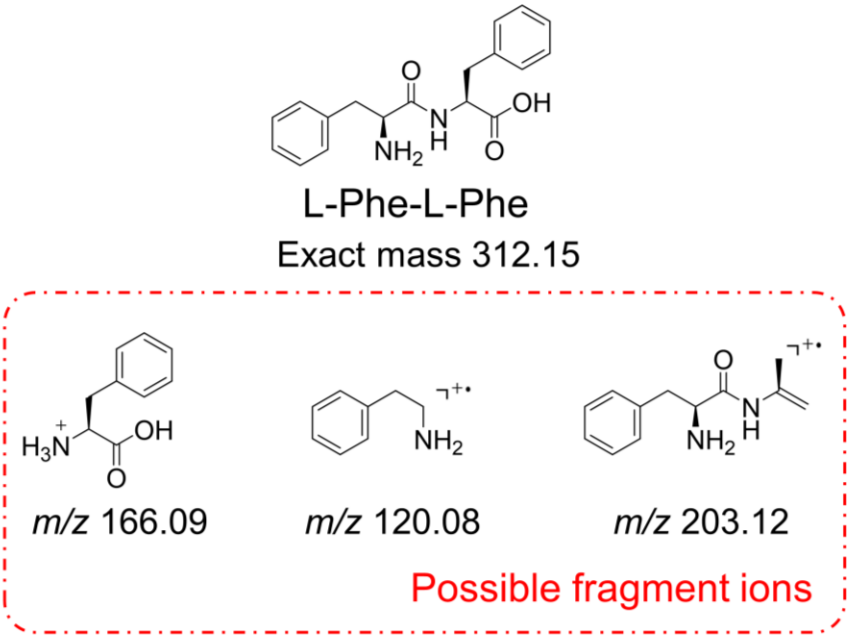

Supplementary Fig. 20. Structural inference of fragment ions produced by L-Phe-L-Phe in the presence of WPMPI-MS with different energy powers.

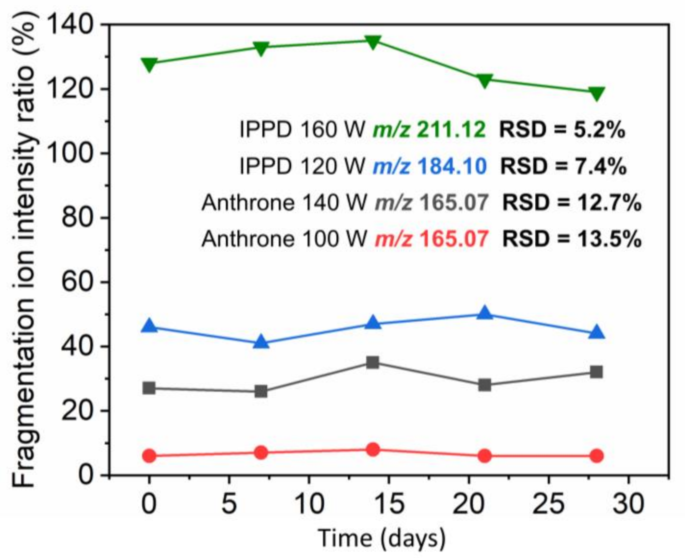

Supplementary Fig. 21. Intensity ratios and of the highest abundance fragment ions of the two compounds at the four energy steps with WPMPI-MS.

Supplementary Note 3

Various application scenarios

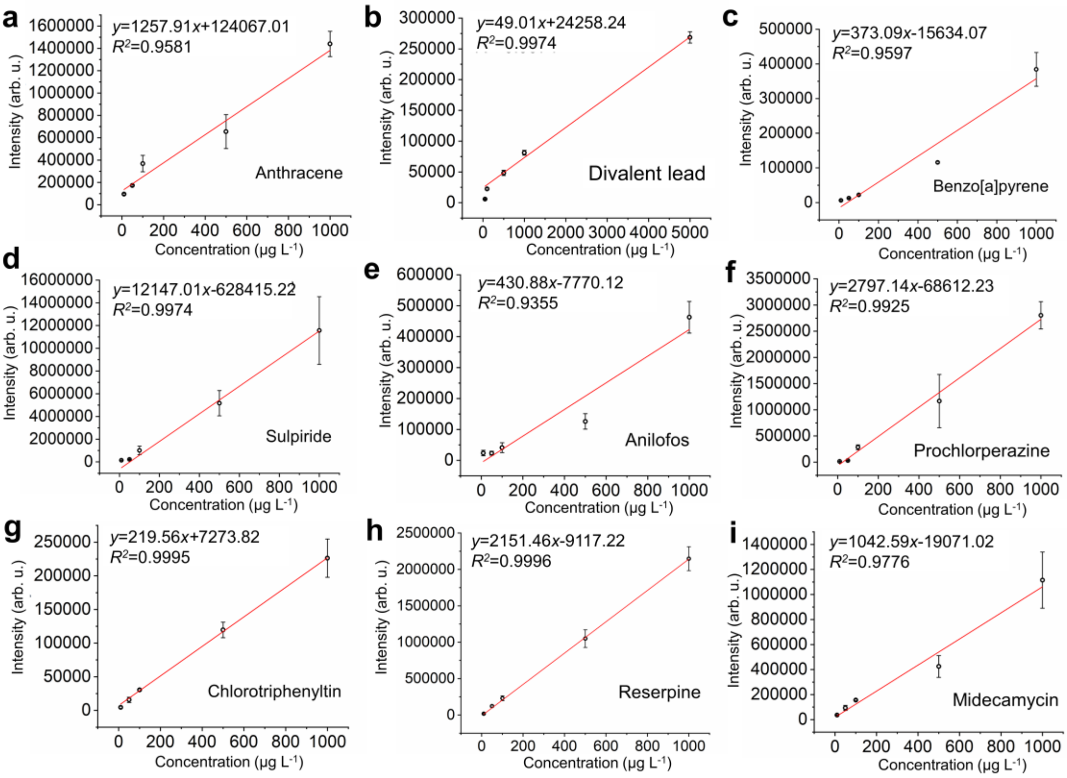

Supplementary Fig. 22. Linear equations for nine toxicological samples **a** anthracene, **b** divalent lead, **c** benzo[a]pyrene, **d** sulpiride, **e** anilofos, **f** prochlorperazine, **g** chlorotriphenyltin, **h** reserpine, and **i** midecamycin under serum extract matrices, the method demonstrated a good R^2 (0.9355-0.9996) over a linear range of 10-5000 μg L^{-1}, where the limit of quantification for inorganic lead was slightly higher than for the remaining analytes. Each value represents the mean ± s.d. ($N = 5$).

Supplementary Table 1. Analytical performance of nine toxicological samples in fast scan mode of WPMPI-MS.

Analyte	Exact mass (g mol^{-1})	Linear equation	Linear range (μg L^{-1})	R^2	LOD (μg L^{-1})	LOQ (μg L^{-1})	RSD n=5
Reserpine	609.2807	y=2151.46x-9117.22	10-1000	0.9996	0.88	2.94	10.4%
Prochlorperazine	404.1558	y=2797.14x-68612.23	10-1000	0.9925	4.82	10.0	14.1%
Midecamycin	814.4583	y=1042.59x-19071.02	10-1000	0.9776	2.98	9.96	12.3%
Sulpiride	342.1482	y=12147.01x-628415.22	10-1000	0.9974	0.98	3.29	16.6%
Anthracene	178.0783	y=1257.91x+124067.01	10-1000	0.9581	1.67	5.60	9.75%
Benzo[a]pyrene	252.0939	y=373.09x-15634.07	10-1000	0.9579	2.15	7.17	7.16%
Chlorotriphenyltin	351.0190	y=219.56x+7273.82	10-1000	0.9995	2.17	7.22	9.31%
Anilofos	368.0305	y=430.88x-7770.12	10-1000	0.9355	2.18	7.26	11.0%
Divalent lead	208.9839	y=49.01x+24258.24	50-5000	0.9974	14.49	48.31	6.21%

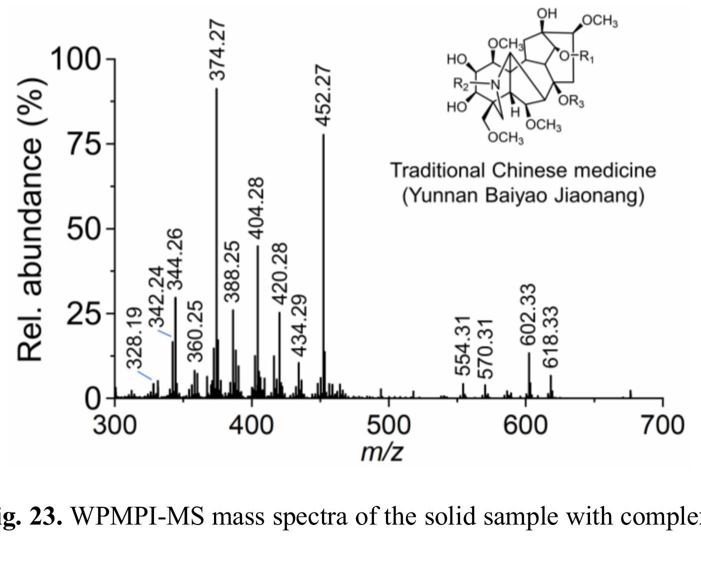

Supplementary Fig. 23. WPMPI-MS mass spectra of the solid sample with complex matrices: Yunnan Baiyao powder (200 μg) in 100-140 W.

Supplementary Table 2. Fourteen high abundant ion signals were screened out and preliminary identified as representative diterpenoids in aconitum.

No.	Compounds	Measured *m*/*z*	Exact *m*/*z*	Mass error (ppm), *n*=5	Formula	Ref.
1	Hetisinone	328.1914	328.1908	1.83	$C_{20}H_{25}NO_3$	2
2	Actaline	342.2432	342.2428	1.17	$C_{22}H_{31}NO_2$	3
3	Heterophyllinine-A	344.2588	344.2583	1.45	$C_{22}H_{33}NO_2$	4
4	Atidine	360.2537	360.2534	0.83	$C_{22}H_{33}NO_3$	2
5	Corumdizine	374.2700	374.2691	2.40	$C_{23}H_{35}NO_3$	3
6	Daphnioldhanine K	388.2501	388.2483	4.63	$C_{23}H_{33}NO_4$	5
7	Aconasutine	404.2805	404.2796	2.23	$C_{24}H_{37}NO_4$	6
8	14-Dehydrotalatisamine	420.2754	420.2745	2.14	$C_{24}H_{37}NO_5$	7
9	Jadwarine-B	434.2921	434.2901	4.61	$C_{25}H_{39}NO_5$	8
10	Vaginaline	452.2682	452.2643	8.62	$C_{24}H_{37}NO_7$	9
11	Pyrochasmaconitine	554.3127	554.3113	2.52	$C_{32}H_{43}NO_7$	10
12	Mithaconitine	570.3072	570.3062	1.75	$C_{32}H_{43}NO_8$	11
13	Sinonapelloinine B	602.3340	602.3324	2.66	$C_{33}H_{47}NO_9$	12
14	8-Deacetylyunaconitine	618.3289	618.3273	2.59	$C_{33}H_{47}NO_{10}$	13

Supplementary Table 3. Compounds detected in landfill leachate by non-target WPMPI-MS.

No.	Name	Ions	Detected *m/z*	Mass error (ppm), *n*=5	Categorization
1	Cu	$[M]^+$	62.9299 (64.9279)	12.7	Metallic
2	Pyrrolidine	$[M+H]^+$	72.0815	9.7	Food additives
3	5-Methylisoxazole	$[M+H]^+$	84.0450	7.1	Industry
4	Rb	$[M]^+$	84.9120 (86.9091)	9.4	Metallic
5	Hexane	$[M-H]^+$	85.1010	-2.3	VOCs
6	2-Pyrrolidinone	$[M+H]^+$	86.0609	9.3	Industry
7	2,3-Butanedione	$[M+H]^+$	87.0449	8.0	VOCs
8	Oxalic acid	$[M-H]^-$	88.9879	1.1	Natural products
9	Pyruvic acid	$[M+H]^+$	89.0231	2.5	Metabolites
10	Aniline	$[M+H]^+$	94.0658	6.4	Industry
11	4-Aminopyridine	$[M+H]^+$	95.0612	8.3	Industry
12	2-Piperidone	$[M+H]^+$	100.0753	3.9	Industry
13	Ethylbenzene	$[M-H]^+$	105.0695	3.8	VOCs
14	Ag	$[M]^+$	106.9053 (108.9051)	8.4	Metallic
15	Benzaldehyde	$[M+H]^+$	107.0498	1.9	VOCs
16	4-Aminobutyric acid	$[M+H]^+$	107.0714	7.4	Natural products
17	2-Acetyl pyrrole	$[M+H]^+$	110.0606	5.9	Food additives
18	Creatinine	$[M+H]^+$	114.0670	7.0	Metabolites
19	1H-Benzotriazole	$[M+H]^+$	120.0556	0.1	Industry
20	Acetophenone	$[M+H]^+$	121.0656	6.6	VOCs
21	2-Acetylpyridine	$[M+H]^+$	122.0604	3.3	Food additives
22	2,6-Dimethylaniline	$[M+H]^+$	122.0967	2.5	Industry
23	Methyl 2-pyrrolecarboxylate	$[M+H]^+$	126.0552	1.6	Industry
24	Naphthalene	$[M]^{+\cdot}$	128.0622	3.1	VOCs
25	3-Methylindole	$[M+H]^+$	132.0813	3.7	Food additives
26	Cs	$[M]^+$	132.9059	7.5	Metallic
27	Quinaldine	$[M+H]^+$	144.0813	3.5	Industry
28	Acetaminophen	$[M+H]^+$	152.0708	1.3	Pharmaceuticals
29	Dopamine	$[M+H]^+$	154.0863	0.1	Metabolites
30	*N, N'*-Methylenebisacrylamide	$[M+H]^+$	155.0817	1.3	Industry
31	2-Phenylpyridine	$[M+H]^+$	156.0809	0.6	Industry
32	*N*-Methylphthalimide	$[M+H]^+$	162.0550	0.1	Industry
33	Xylan	$[M-H]^-$	165.0403	1.0	Natural products
34	Methyl 2-(methylamino)benzoate	$[M+H]^+$	166.0864	0.6	Food additives
35	4-Aminobiphenyl	$[M+H]^+$	170.0966	1.2	Industry

36	Cotinine	$[M+H]^+$	177.1025	1.7	Metabolites
37	Diethyl Itaconate	$[M+H]^+$	187.0948	9.1	Industry
38	Galactonic acid	$[M-H]^-$	195.0509	0.1	Industry
39	Caffeine	$[M+H]^+$	195.0876	-0.5	Metabolites, food
40	1,1-Dibutoxytrimethylamine	$[M+H]^+$	204.1952	2.9	Industry
41	Pb	$[M]^{+\cdot}/[M+H]^+$	207.9781/208.9850	7.2	Metallic
42	Laurophenone	$[M+H]^+$	261.2216	1.2	Industry
43	2,4,6-Tri-tert-butylphenol	$[M+H]^+$	263.2373	1.5	Industry
44	Heptadecanoic acid	$[M+H]^+$	271.2622	3.5	Industry
45	Alpha-Linolenic acid	$[M+H]^+$	279.2312	2.4	Industry
46	Octadecanamide	$[M+H]^+$	284.2935	4.5	Industry
47	Testosterone	$[M+H]^+$	289.2168	2.1	Pharmaceuticals
48	Perfluorobutane sulfonyl fluoride	$[M-H]^-$	298.9427	-1.0	Industry
49	*N*-Methyldidecylamine	$[M+H]^+$	312.3608	5.4	Industry
50	Phytosphingosine	$[M+H]^+$	318.2993	3.1	Pharmaceuticals
51	Valaciclovir	$[M+H]^+$	325.1614	-2.6	Pharmaceuticals
52	Sinensetin	$[M+H]^+$	373.1284	-0.6	Natural products
53	Perfluorohexane sulfonyl fluoride	$[M-H]^-$	398.9360	-1.5	Industry
54	Hexamethylquercetagetin	$[M+H]^+$	403.1378	2.7	Natural products
55	Eplerenone	$[M+H]^+$	415.2104	1.1	Pharmaceuticals
56	Tetracycline	$[M+H]^+$	445.1598	-1.6	Pharmaceuticals

*Inside parentheses represent the highest isotopic peaks of metal ions.

Supplementary Note 4

Supplementary Table 4. Comparison of ionizable analyte types between WPMPI and conventional ion sources

Analyte type	EI	ESI	ICP	APCI	MALDI	WPMPI
Polar	○	○	-	□	○	○
Non-polar	○	×	-	○	○	○
Volatile	○	○	-	○	×	○
Non-volatile	×	○	-	□	○	○
Metal element	×	×	○	×	×	○
In-source cleavage	○	×	-	□	□	○

* ○ Detectable; × Undetectable; □ Mediocre results.

Microwave safety issues

For microwave safety, we applied perfect shielding to both the power module and the transmission line, and used professional RF connecting lines to place microwave leakage (**Supplementary Fig. 24a**). In addition, we tested the microwave radiation (HT-M2 Microwave Leakage Detector, Dongguan Xintai Instrument Co., Ltd.) in the working condition of the device, and the results are shown in **Supplementary Fig. 24b-d**. In the main part of the rectangular tube, even if the detector is close to the surface, the measured microwave radiation value is only about 1.21 mW cm^{-2}, making it nearly undetectable beyond 10 cm. In the front section of the plasma, the microwave leakage at 3 cm is approximately 3.38 mW cm^{-2}, which is lower than the standards of various countries (Chinese National Standard 5 mW cm^{-2}, and United States Standard 10 mW cm^{-2}, operating position), and the attenuation outside 10 cm is 0.78 mW cm^{-2}, which is hardly detectable outside 20 cm. During general operation, the operator does not need to be within 20 cm of the plasma front.

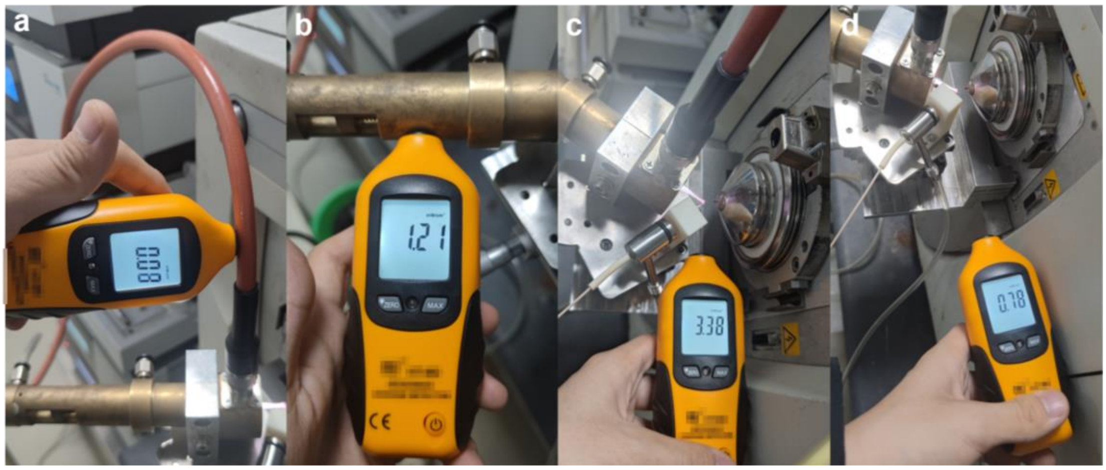

Supplementary Fig. 24. The microwave radiation levels were measured at various locations during the operation of WPMPI: **a** connecting line, **b** stick close to the main body, **c** 3 cm from the plasma tip, **d** 10 cm from the plasma tip.

Supplementary Note 5

Experimental section

The microwave plasma generation module and nebulizer are placed orthogonally towards the ambient mass spectrometry inlet. A digital control board (purchase from Feiyi Technology, Hangzhou, China) regulates the output power and timing of the plasma microwave power supply, which can be used including common microcontrollers, arbitrary wave generators, and NI acquisition cards. The configured solution or gas sample is added to the injector and then ejected from the nebulizer into the plasma beam region where ionization occurs. The nebulizer should be a quartz nebulizer to avoid damage to the equipment from excessive temperatures when the plasma power is increased. All experiments are supported by high-resolution mass spectrometry.

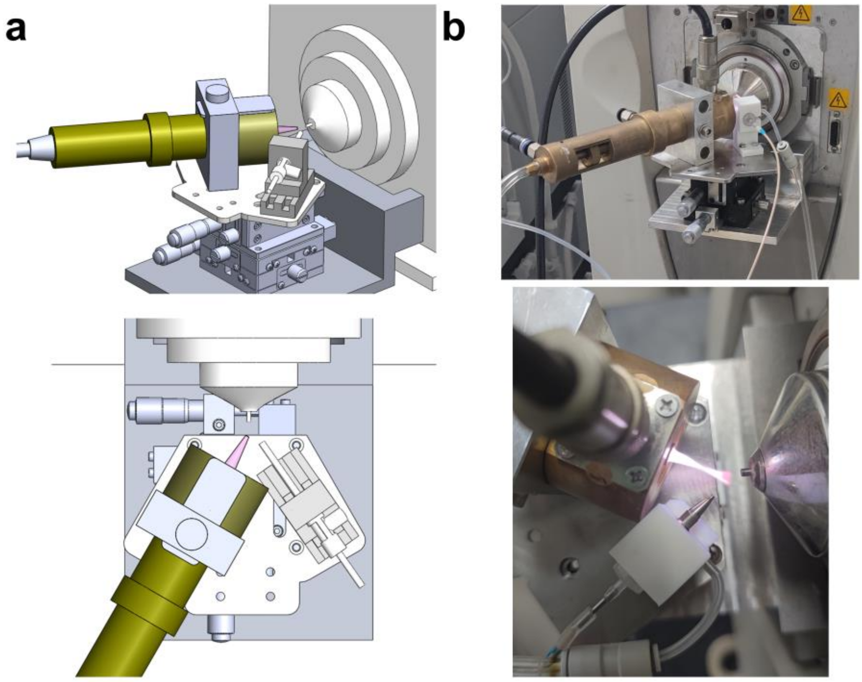

Supplementary Fig. 25. Schematic **a** and physical **b** drawings for modeling key components of WPMPI-MS.

Design Details

To realize the fast and stable switching of the microwave energy, firstly, the size of the resonant cavity is increased compared with the microwave plasma device used by the previous work[14,15], the detailed diameter of the microwave plasma tube is now as follow: outer tube (44 mm outer diameter (O.D.), 21 mm inner diameter (I.D.)), central tube (7 mm O.D., 5 mm I.D.) and intermediate tube (3.0 mm O.D.,

1.5 mm I.D.). The motion distance of the tuning end plane is 40 mm. The larger size results in better heat dissipation and the relatively larger power range supported, also increases the travel range for impedance matching adjustment. The choice of working gas, argon, is critical. Compared to helium, nitrogen or air, argon has the lowest discharge power. In this design, the microwave power of about 40 W can activate the relatively stable plasma torch, which leads to an ionization zone with relatively low temperature and energy.

The adjustment of the resonant cavity is also crucial. After the microwave plasma work, the end plane in the rectangular tube needs to be adjusted. Real-time monitoring of reflected power by means of oscilloscopes in the microwave power supply to minimize the value of reflected power for impedance matching. The actual test results show that when the tuning end face and resonant cavity end face are 29 mm apart, the microwave reflection is minimal, resulting in a microwave energy utilization of more than 95%. To increase the stability of the microwave power supply's fast power switching operation, a circulator is added to the power supply output. The reflected power during plasma operation is guided to the forward output through the circulator, avoiding the direct impact of the reflected power on the microwave power supply to increase the stability of the power supply's forward output power.

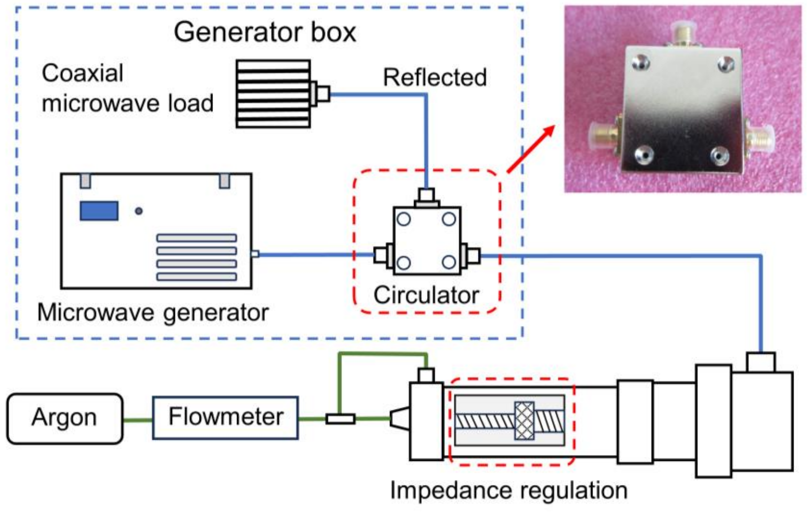

Supplementary Fig. 26. The specific design drawing of the main part of WPMPI.

Supplementary Table 5. Mass error of the analytes

Chemicals	Ions	Exact *m*/*z*	Measured *m*/*z*	Mass error (ppm), *n*=5
**Methyl salicylate**	$[M+H]^+$	153.0551	153.0546	3.27
**Methyl salicylate**	$[M]^{\cdot +}$	152.0472	152.0473	-0.66
**Divalent lead**	PbH^+	208.9839	208.9835	1.91
**Reserpine**	$[M+H]^+$	609.2807	609.2793	2.30
**Divalent lead**	$PbOH^+$	224.9788	224.9795	-3.11
**Gly-His-Lys**	$[M+H]^+$	341.1932	341.1918	4.10
**Bradykinin**	$[M+H]^+$	1060.569	1060.568	1.04
**Dermaseptin**	$[M+H]^{2+}$	1728.437	1728.442	-2.95
**Melezitose**	$[M+H]^+$	505.1775	505.1763	2.38
**ApC**	$[M-H]^-$	571.1306	571.1308	-0.35
**Benzo[a]pyrene**	$[M]^{\cdot +}$	252.0939	252.0941	-0.79
**Anthracene**	$[M]^{\cdot +}$	178.0783	178.0777	3.37
**Sulpiride**	$[M+H]^+$	342.1482	342.1474	2.34
**Anilofos**	$[M+H]^+$	368.0305	368.0301	1.09
**Midecamycin**	$[M+H]^+$	814.4583	814.4581	0.25
**Prochlorperazine**	$[M+H]^+$	404.1558	404.1556	0.49
**Chlorotriphenyltin**	$[M-Cl]^+$	351.019	351.0193	-0.85
**Pyrene**	$[M]^{\cdot +}$	202.0783	202.078	1.48
**Chrysene**	$[M]^{\cdot +}$	228.0939	228.0932	3.07
**Tributyltin hydride**	$[M-H]^+$	291.1129	291.1145	-5.50
**Ethyl acetate**	$[M-H]^+$	87.0441	87.0444	3.45
**Anthraquinone**	$[M+H]^+$	209.0597	209.0598	-0.48
**2-Pentanone**	$[M+H]^+$	87.0804	87.0809	5.74
**4-Phenylbutylamine**	$[M+H]^+$	150.1277	150.1281	2.66
**PFOS-K**	$[M-H]^-$	498.9302	498.9317	3.01
**PFBS**	$[M-H]^-$	298.9430	298.9443	4.35

**PFOA**	$[M-H]^{-}$	412.9664	412.9681	4.12
**Chromium**	$[M]^{\cdot+}$	113.9028	113.9032	3.51
**Bromine**	Br^{-}	78.9183	78.9173	12.67
**Hexabromobenzene**	$[M-Br+O]^{-}$	486.5831	486.572	-22.81

Supplementary References

1. Zhu, S. Z., Zhang, L., Zhang, J. & Guo, Y. L. Arc Plasma-Based Dissociation Device: Fingerprinting Mass Spectrometric Analysis Realized at Atmospheric Condition. *Anal. Chem.* **92**, 14633-14639, doi:10.1021/acs.analchem.0c03127 (2020).
2. Ahmad, H.; Ahmad, S.; Shah, S. A. A.; Latif, A.; Ali, M.; Khan, F. A.; Tahir, M. N.; Shaheen, F.; Wadood, A.; Ahmad, M. Antioxidant and anticholinesterase potential of diterpenoid alkaloids from Aconitum heterophyllum. *Bioorgan. Med. Chem.* **25**, 3368-3376 (2017).
3. Tel'nov, V. A.; Usmanova, S. K.; Abdullaev, N. D. Structure of acsinatine. *Chem. Nat. Compd.* **29**, 346-348 (1993).
4. Nisar, M.; Obaidullah; Ahmad, M.; Wadood, N.; Lodhi, M. A.; Shaheen, F.; Choudhary, M. I. New diterpenoid alkaloids from Aconitum heterophyllum Wall: Selective butyrylcholinestrase inhibitors. *J. Enzyme Inhib. Med. Chem.* **24**, 47-51 (2009).
5. Mu, S. Z.; Wang, J. S.; Yang, X. S.; He, H. P.; Li, C. S.; Di, Y. T.; Wang, Y.; Zhang, Y.; Fang, X.; Huang, L. J.; Hao, X. J. Alkaloids from Daphniphyllum oldhami. *J. Nat. Prod.* **71**, 564-569 (2008).
6. Mericli, A. H.; Mericli, F.; Becker, H.; Ulubelen, A. A new prodelphinine type alkaloid from *Aconitum* nasutum. *Turk. J. Chem.* **20**, 164-167 (1996).
7. Salimov, B. T.; Yunusov, M. S.; Yunusov, S. Y. Alkaloids of delphinium biternatum. *Khim. Prir. Soedin.***14**, 84-88 (1978).
8. Ahmad, H.; Ahmad, S.; Ali, M.; Latif, A.; Shah, S. A. A.; Naz, H.; Rahman, N. U.; Shaheen, F.; Wadood, A.; Khan, H. U.; Ahmad, M. Norditerpenoid alkaloids of Delphinium denudatum as cholinesterase inhibitors. *Bioorg. Chem.* **78**, 427-435 (2018).
9. Qing, P. J.; Wei, L. S. The diterpenoid alkaloids from Aconitum-Scaposum Var Vaginatum. *Heterocycles* **24**, 877-879 (1986).
10. Jian-Min, Y.; Xu, J.; Yao-Zu, C. C19-diterpenoid alkaloids of *Aconitum* kongboense. *Phytochemistry* **35**, 829-831 (1994).
11. Pelletier, S. W.; Mody, N. V.; Sawhney, R. S.; Bhattacharyya, J. Application of carbon-13 MR spectroscopy to the structural elucidation of C19-diterpenoid alkaloids from *Aconitum* and *Delphinium* species. *Heterocycles* **7**, 327 (1977).
12. Wang, F.-P.; Chen, Q.-H., The C19-Diterpenoid Alkaloids. In *The Alkaloids: Chemistry and*

Biology, Cordell, G. A., Ed. Academic Press: 2010; Vol. 69, pp 1-577.

13. Donglin, C.; Xixian, J.; Qiaohong, C.; Fengpeng, W. New C(19)-diterpenoid alkaloids from the roots of *Aconitum* transsecutum. *Acta Chim. Sinica* **61**, 901-906 (2003).
14. Miao, M., Zhao, G., Wang, Y., Xu, L., Dong, J., Cheng, P. Spray-inlet microwave plasma torch ionization tandem mass spectrometry for the direct detection of drug samples in liquid solutions. *Rapid Commun. Mass Spectrom* **31**, 2092–2100 (2017)
15. Zhang, T., Zhou, Wei., Jin, W., Zhou, J., Handberg, E., Zhu, Z., Chen, H., Jin, Q. Direct desorption/ionization of analytes by microwave plasma torch for ambient mass spectrometric analysis. *J. Mass Spectrom.* **48,** 669–676 (2013)
