# Supplementary material for: Wide-energy programmable microwave plasma-ionization for high-coverage mass spectrometry analysis
Source: Nat Commun. 2024 Jul 18;15:6075. doi: 10.1038/s41467-024-50322-z (PMC11258349; doi:10.1038/s41467-024-50322-z)
Supplement: Supplementary file 3 — Reporting Summary [file 41467_2024_50322_MOESM3_ESM.pdf]

nature portfolio

Corresponding author(s): Xiaozhi Wang, Hongru Feng, Yuanjiang Pan

Last updated by author(s): Jun 14, 2024

Reporting Summary

Nature Portfolio wishes to improve the reproducibility of the work that we publish. This form provides structure for consistency and transparency in reporting. For further information on Nature Portfolio policies, see our Editorial Policies and the Editorial Policy Checklist.

Statistics

For all statistical analyses, confirm that the following items are present in the figure legend, table legend, main text, or Methods section.

n/a	Confirmed	
☐	☒	The exact sample size (n) for each experimental group/condition, given as a discrete number and unit of measurement
☐	☒	A statement on whether measurements were taken from distinct samples or whether the same sample was measured repeatedly
☒	☐	The statistical test(s) used AND whether they are one- or two-sided<br>*Only common tests should be described solely by name; describe more complex techniques in the Methods section.*
☐	☒	A description of all covariates tested
☐	☒	A description of any assumptions or corrections, such as tests of normality and adjustment for multiple comparisons
☐	☒	A full description of the statistical parameters including central tendency (e.g. means) or other basic estimates (e.g. regression coefficient) AND variation (e.g. standard deviation) or associated estimates of uncertainty (e.g. confidence intervals)
☒	☐	For null hypothesis testing, the test statistic (e.g. F, t, r) with confidence intervals, effect sizes, degrees of freedom and P value noted<br>*Give P values as exact values whenever suitable.*
☒	☐	For Bayesian analysis, information on the choice of priors and Markov chain Monte Carlo settings
☐	☒	For hierarchical and complex designs, identification of the appropriate level for tests and full reporting of outcomes
☐	☒	Estimates of effect sizes (e.g. Cohen's d, Pearson's r), indicating how they were calculated

Our web collection on statistics for biologists contains articles on many of the points above.

Software and code

Policy information about availability of computer code

Data collection	Experiments were carried out using a Thermo LTQ Orbitrap mass spectrometer (Thermo Fisher Scientific, Waltham, MA). Data collection was performed using the Xcalibur® software (version 2.2 SP1.48; TFS, SanJose, CA)
Data analysis	Data analysis was performed using the Xcalibur® software (version 2.2 SP1.48; TFS, SanJose, CA), FreeStyle v1.8 (Thermo Scientific, USA), and MSFinder v3.6.

For manuscripts utilizing custom algorithms or software that are central to the research but not yet described in published literature, software must be made available to editors and reviewers. We strongly encourage code deposition in a community repository (e.g. GitHub). See the Nature Portfolio guidelines for submitting code & software for further information.

Data

Policy information about availability of data

All manuscripts must include a data availability statement. This statement should provide the following information, where applicable:

- Accession codes, unique identifiers, or web links for publicly available datasets
- A description of any restrictions on data availability
- For clinical datasets or third party data, please ensure that the statement adheres to our policy

he data that support the findings of this study are available within the paper and the Supplementary Information. The raw data are available from Figshare (https://doi.org/10.6084/m9.figshare.24590739). Other relevant data are available from the corresponding authors on request. No clinical datasets or third party data.

Research involving human participants, their data, or biological material

Policy information about studies with human participants or human data. See also policy information about sex, gender (identity/presentation), and sexual orientation and race, ethnicity and racism.

Reporting on sex and gender	The research did not involve human participants, their data or biological materials.
Reporting on race, ethnicity, or other socially relevant groupings	The research did not involve human participants, their data or biological materials.
Population characteristics	The research did not involve human participants, their data or biological materials.
Recruitment	The research did not involve human participants, their data or biological materials.
Ethics oversight	The research did not involve human participants, their data or biological materials.

Note that full information on the approval of the study protocol must also be provided in the manuscript.

Field-specific reporting

Please select the one below that is the best fit for your research. If you are not sure, read the appropriate sections before making your selection.

☐ Life sciences ☐ Behavioural & social sciences ☒ Ecological, evolutionary & environmental sciences

For a reference copy of the document with all sections, see nature.com/documents/nr-reporting-summary-flat.pdf

Ecological, evolutionary & environmental sciences study design

All studies must disclose on these points even when the disclosure is negative.

Study description	Non-targeted analysis of contaminants in landfill leachates.
Research sample	The landfill leachate is taken from the waste transfer station at Wuxing Avenue, Huzhou, Zhejiang, China.
Sampling strategy	A raw landfill leachate sample was collected in May 2024 from a domestic landfill in northern Zhejiang, China, using polypropylene vials to collect leachate from the tap. The samples were transported to our laboratory and stored at -20 °C before preparation.
Data collection	C.F tested and collected data using Orbitrap Exploris 120 (Thermo Scientific, Bremen, Germany) mass spectrometry in May 2024.
Timing and spatial scale	Data collection began on May 8 and ended on May 14, during which three tests were conducted, each recording 2-3 sets of data. A single leachate sample was used, so no sampling was required.
Data exclusions	No data were excluded.
Reproducibility	The test was attempted three times with approximate and repeatable results and no failed experiments.
Randomization	A single leachate sample was used, so no allocation was required.
Blinding	A single leachate sample was used, so blinding was no required.

Did the study involve field work? ☐ Yes ☒ No

Reporting for specific materials, systems and methods

We require information from authors about some types of materials, experimental systems and methods used in many studies. Here, indicate whether each material, system or method listed is relevant to your study. If you are not sure if a list item applies to your research, read the appropriate section before selecting a response.

Materials & experimental systems

n/a	Involved in the study
☒	☐ Antibodies
☒	☐ Eukaryotic cell lines
☒	☐ Palaeontology and archaeology
☒	☐ Animals and other organisms
☒	☐ Clinical data
☒	☐ Dual use research of concern
☒	☐ Plants

Methods

n/a	Involved in the study
☒	☐ ChIP-seq
☒	☐ Flow cytometry
☒	☐ MRI-based neuroimaging

Plants

Seed stocks	No plant material used.
Novel plant genotypes	No plant material used.
Authentication	No plant material used.
